# Vegetation type and grazing intensity jointly shape grazing effects on grassland biodiversity

**DOI:** 10.1002/ece3.4508

**Published:** 2018-10-03

**Authors:** Péter Török, Károly Penksza, Edina Tóth, András Kelemen, Judit Sonkoly, Béla Tóthmérész

**Affiliations:** ^1^ MTA‐DE Lendület Functional and Restoration Ecology Research Group Debrecen Hungary; ^2^ Department of Ecology University of Debrecen Debrecen Hungary; ^3^ Department of Botany Institute of Botany and Ecophysiology Szent István University Gödöllő Hungary; ^4^ MTA TKI MTA's Post Doctoral Research Program Budapest Hungary; ^5^ MTA‐DE Biodiversity and Ecosystem Services Research Group Debrecen Hungary

**Keywords:** functional diversity, leaf traits, overgrazing, plant traits, prairie, steppe

## Abstract

In the Palaearctic steppe zone, overgrazing was identified as one of the key drivers of declining grassland biodiversity, which underlines the necessity of the functional evaluation of increased grazing pressure on grassland vegetation. We tested the following hypotheses: (a) The effect of grazing intensity on species and functional diversity is strongly dependent on grassland type. (b) The magnitude of diet selectivity of grazers decreases with increasing grazing intensity. (c) Increasing grazing intensity increases evenness and functional evenness of the subjected grasslands. We analyzed vegetation patterns in four types of grasslands (Dry alkali short‐grass steppes, Dry loess steppes, Non‐alkali wet and Alkali wet grasslands) along an intensity gradient of beef cattle grazing at 73 sites in Hungary. Species richness, Shannon diversity, evenness, and four leaf traits were analyzed. We calculated community‐weighted means for each single trait, and multi‐trait functional richness, functional evenness, and divergence for all leaf traits. All species and functional diversity metrics were significantly affected by the grassland type, except leaf dry matter content. The effect of interaction between grazing intensity and grassland type was also significant for functional richness, functional evenness, community‐weighted means of leaf area, and for species richness and evenness. An upward trend of specific leaf area was detected in all grasslands with the highest scores for the overgrazed sites, but the change was also grassland type dependent. The detected trend suggests that with increased intensity the overall selectivity of grazing decreased. We found that evenness was affected but functional evenness was not affected by grazing intensity. Functional evenness scores were more related to the grassland type than to changes in grazing intensity, and displayed a high variability. We stress that one‐size‐fits‐all strategies cannot be recommended and actions should be fine‐tuned at least at the level of grassland type.

## 1 INTRODUCTION

Grasslands and other open habitats are usually maintained by livestock grazing (Evans et al., 2015). In historical times grazing was provided by free ranging large wild grazers, but with the increase of human influence in the landscape, this was gradually replaced mostly by herded livestock (Bakker et al., 2004; Pykäla, 2000). The regular low‐intensity livestock grazing maintained or created high nature value farming systems with remarkably high biodiversity (Isselstein, Griffith, Pradel, & Venerus, 2007; Tälle et al., 2016; Török et al., 2016). In the past century, the traditional management systems in many agro‐ecosystems were replaced by intensive grazing systems characterized by the application of inappropriate grazers and/or high stocking rates (Metera, Sakowski, Słoniewski, & Romanowicz, 2010; Rook et al., 2004).

Globally, grazing patterns are rapidly changing. In some regions such as European mountain ranges, the cessation of grazing is typical, while in most lowland regions of Australia, Africa, Asia, South America, and the Mediterranean, overgrazing has become typical (Abu Hammad & Tumeizi, 2012; Evans et al., 2015; Lu et al., 2017; Rowntree, Duma, Kakembo, & Thornes, 2004; Vetter & Bond, 2012). In several regions within the vast Palaearctic steppe zone, which stretches from Eastern Europe to Northeast China, overgrazing, alongside land conversion, became the most important driver of declining grassland biodiversity in the last few decades (Wesche et al., 2016).

Livestock grazing is one of the most important drivers of biodiversity, and the most important land‐use type influencing the ecosystem properties of subjected habitats (Eldridge, Poore, Ruiz‐Colmenero, Letnic, & Soliveres, 2016; Golodets, Kigel, & Sternberg, 2010). Grazing reduces biomass, plant cover, litter and thus increases available regeneration niches for gap strategists (Hofmann & Isselstein, 2004). It directly shifts the composition of plant communities by diet selection, and also changes the structural and compositional heterogeneity by the suppression of competitors and by changing the light availability on the soil surface (Rook et al., 2004). Grazing also contributes to species dispersal processes by endo‐ and ectozoochory (Ozinga et al., 2009). By trampling, grazing animals alter the surface structure and functioning of the soil by increasing compactness and reducing soil porosity (Lunt, Eldridge, Morgan, & Witt, 2007). The damage of the soil structure and the expansion of open surfaces may cause an increased rate of erosion and deflation (Lu et al., 2017). Grazing can also influence the organic matter content of the soil affecting decisive processes of mineralization and decomposition (Peco, Navarro, Carmona, Medina, & Marques, 2017; Waters, Orgill, Melville, Toole, & Smith, 2017; Zhou et al., 2017).

Recently, the functional analysis of grazing effects on the vegetation based on specific plant traits has become a “hot topic” (Kechang, He, Zhang, & Lechowicz, 2015; Komac, Pladevall, Domènech, & Fanlo, 2015; Teuber, Hölzel, & Fraser, 2013). The trait‐based functional diversity approach can reveal the functioning of the ecosystem and mechanisms beyond the changes of taxonomic diversity and composition; thus, helping to explain dynamic changes in ecosystems (Carmona, Mason, Azcárate, & Peco, 2015; Villéger, Mason, & Mouillot, 2008). Despite the huge global extent of grazing and its importance for food production, land management, restoration and conservation of natural habitats, detailed functional analyses on the effects of grazing are lacking (De Bello, Lepš, & Sebastiá, 2005; Díaz Barradas, García Novo, Collantes, & Zunzunegui, 2001).

The most important factors which determine the effects of grazing on ecosystem properties are summarized by Eldridge et al. (2016): (a) type of herbivore, (b) intensity of grazing pressure, (c) level of plant productivity, and (d) evolutionary history of grazing. In our study we focused on factors (b) and (c), analyzing beef cattle grazing in four grassland types within landscapes with similar evolutionary grazing history, thereby controlling for factors (a) and (d).

We analyzed trait‐based vegetation patterns in four types of grasslands with different productivity along a grazing intensity gradient. We specifically tested three hypotheses related to grazing intensity, grassland types and to their interaction. Former research suggested that the grazing behavior of livestock is strongly influenced by the biomass production (Mládek, Hejcman, Hejduk, Duchoslav, & Pavlů, 2011) and the species composition and richness (Liu et al., 2015) of the subjected habitat. Thus, we expected that grazing effects will be markedly different in grassland types with different species composition, biomass, and diversity. We tested the (a) *Habitat‐dependent effects of intensity* hypothesis, and we expected that the effect of grazing intensity on species and functional diversity is strongly grassland type dependent.

Cattle is a less selective grazer compared to sheep, and displays higher selectivity for community dominants (i.e., graminoids), especially in low diversity communities (Rook et al., 2004). However, former research also suggests that grazers’ selectivity and feeding strategy may change with increasing stocking rates and/or between communities (Liu et al., 2015), and diet selectivity targets vegetation or species with higher nutritive value (Carmona et al., 2012). In case of low stocking rates, high quality fodder is available in sufficient amount for all grazers in most communities, which in general enables grazers to express higher selectivity for high quality fodder, especially in diverse communities (Liu et al., 2015). With the increase in stocking rates, the livestock is forced also to select lower quality fodder because of the limited availability of high quality fodder (Mládek et al., 2013). This decreasing selectivity might be expressed indirectly in the increase of specific leaf area values and in the decrease of leaf dry matter contents (Tóth et al., 2018). Thus, we tested the (b) *Intensity‐dependent selectivity* hypothesis, and we assumed that the magnitude of diet selectivity of grazers decreases with increasing grazing intensity, which is expressed in the increase of specific leaf area and the decrease of leaf dry matter content values.

As cattle grazing is mostly targeted to dominant species in the community (Rook et al., 2004). We expect that with the increase in grazing intensity the abundance of characteristic graminoids decreases and the abundance of subordinated species increases (Liu et al., 2015). Former research also supports that cattle grazing is less selective for forbs, sustaining a higher species richness compared to sheep grazing (Jerrentrup, Seither, Petersen, & Isselstein, 2015; Metera et al., 2010). Thus, we tested in our research the (c) *Intensity‐dependent evenness* hypothesis, and assumed that increasing grazing intensity increases the evenness and functional evenness (FEve) of the grasslands.

## 2 MATERIALS AND METHODS

### 2.1 Study setup

**Figure 1:**
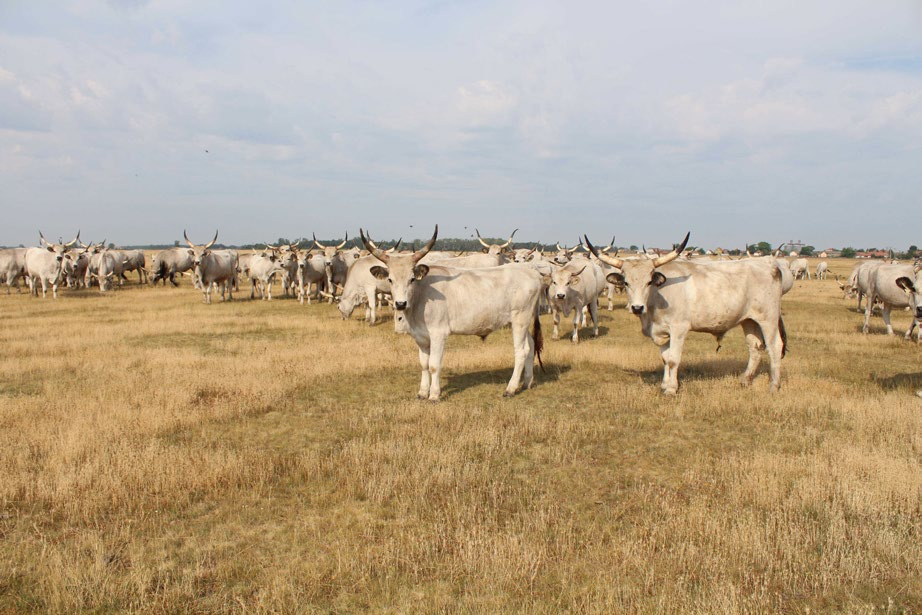
Dry alkali short‐grass steppes grazed with Hungarian gray cattle in high intensity. Photo by A. Kelemen

In total, 73 sites grazed by beef cattle, predominantly Hungarian gray cattle, were selected for the study (Figure 1). The sites were selected to cover four grassland types (dry alkali short‐grass steppes, dry loess steppes, non‐alkali wet grasslands, alkali wet grasslands) typically grazed by beef cattle and to cover four levels of grazing intensity (low‐intensity grazing = <1.0 animal unit (AU in the following) per ha; medium intensity = 1.0–2.5 AU/ha; high intensity = 3.0–8.0 AU/ha; and overgrazed = ≥20.0 AU/ha). Grazing intensities were relatively constant at each site in the last five consecutive years before the given site was sampled. We selected in total 622 2 × 2 m plots for which vascular plant species percentage cover records were available. The vegetation records originated from a grazing database collected by the authors of the present paper (K. Penksza et al., unpublished). It contains records from the years between 1997 and 2016 using a standardized methodology for cover estimate. Vegetation was recorded at the peak of biomass production in each grassland type (between late April, and end of June considering site, grassland type and year). The detailed list of sites, plot numbers and sampling years are in Supporting Information (Table S1). Most records were collected in the period of 2002–2016 (only one site was sampled in 1997) and 2012 was the only dry year (affecting five sites out of 73). Most of the sample years (68 sites) were characterized by average or higher than the hundred‐year average precipitation. Thus, short‐lived species were well‐represented also in the driest plant communities (Supporting Information Table S1).

### 2.2 Studied grasslands

#### 2.2.1 Dry alkali short‐grass steppes

**Figure 2:**
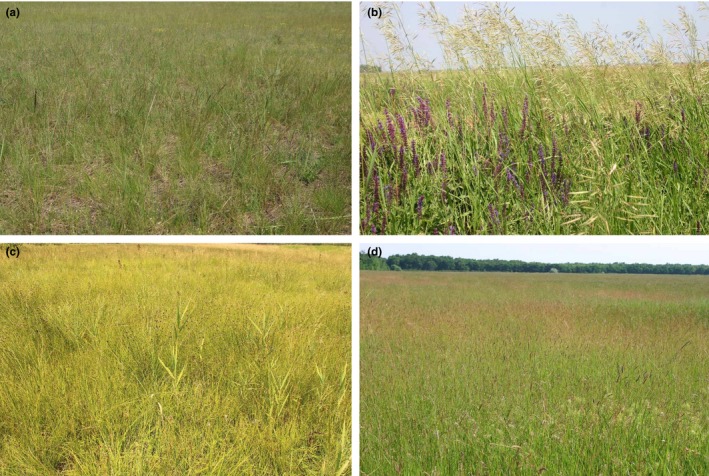
The physiognomy of the studied grassland types. Notations: (a) Dry alkali short‐grass steppes, (b) Dry loess steppes, (c) Non‐alkali wet grassland, (d) Wet grassland. Photos a—K. Penksza; b, c and d—A. Kelemen

Short‐grass steppes are characterized by high cover of short‐growing fescue *Festuca pseudovina*. The cover of *Festuca pseudovina* Hack. is in general 40–70% (Kelemen et al., 2015). Characteristic subordinated species are *Achillea collina*,* A. setacea*,* Artemisia santonicum*,* Bupleurum tenuissimum*,* Cerastium dubium*,* Gypsophila muralis*,* Inula britannica*,* Limonium gmelinii* subsp. *hungaricum*,* Podospermum canum*,* Plantago lanceolata*, and *Trifolium* species (*T. angulatum*,* T. retusum*,* T. striatum* and *T. strictum*). The total biomass (including both green biomass and litter) measured at the peak of the production, in general, does not exceed 200 g/m^2^ (Kelemen, Török, Valkó, Miglécz, & Tóthmérész, 2013). The soils of these grassland types are nutrient‐poor solonchak and solonetz characterized by a low to moderate salt content (Török, Kapocsi, & Deák, 2011). The dry alkali short‐grass steppes are frequently managed by low‐intensity grazing by cattle or sheep. In the heavily grazed stands *Bromus hordeaceus, Cynodon dactylon, Elymus repens*,* Polygonum aviculare* and *Tripleurospermum perforatum* are common **(**Török, Kapocsi, & Deák, 2011; Figure 2a).

#### 2.2.2 Dry loess steppes

The dry loess steppes are species‐rich communities; the characteristic graminoids are *Agropyron cristatum, Bromus inermis*,* Festuca rupicola*,* Koeleria cristata*,* Poa angustifolia,* and *Stipa capillata* (Török, Kelemen, et al., 2011). They harbor forb species such as *Phlomis tuberosa, Salvia austriaca*,* S. nemorosa, Thalictrum minus* and *Thymus glabrescens* in relatively high cover. They are generally managed by low‐intensity cattle grazing or mowing. Heavily grazed stands are characterized by a high cover of grazing tolerant grasses (*Cynodon dactlyon*,* F. rupicola*,* P. angustifolia*), sedges (*Carex stenophylla*) and spiny forbs (*Eryngium campestre, Ononis spinosa*) (Török, Kelemen, et al., 2011; Figure 2b). The total biomass (including both green biomass and litter) measured at the peak of the production typically ranges between 380 and 600 g/m^2^ (Kelemen et al., 2013). The soil types are various forms of nutrient‐rich chernozem soils characterized by a neutral soil pH.

### 2.3 Non‐alkali wet grasslands

This vegetation made up of tall‐growing graminoid species is typically found along rivers and floodplains and places with spring surface waters, where soil pH ranges from the slightly acidic to slightly basic. The characteristic graminoid species are *Deschampsia caespitosa* and *Agrostis stolonifera*. In other types, *Carex acutiformis* and *C. riparia* are very common. Further subordinated species are other sedge species such as *C. distans, C. panicea* or *C. vulpina,* grass species such as *Holcus lanatus*, and forb species such as *Ranunculus acris* and *R. repens* (Borhidi, Kevey, & Lendvai, 2012; Figure 2c). The total biomass (including both green biomass and litter) measured at the peak of the production typically ranges between 600 and 900 g/m^2^ (Penksza et al., unpublished). The soils are wet and compact meadow soils (gleysoils, fluvisoils, or vertisoils) with highly varying contents of nutrients but with no salt contents.

### 2.4 Alkali wet grasslands

Grasslands within this grassland type are characterized by tall‐growing grasses such as *Agrostis stolonifera*,* Alopecurus pratensis*,* Beckmannia eruciformis*,* Elymus repens* and *Glyceria fluitans*. In some stands, high cover of *Bolboschoenus maritimus* and *Phalaris arundinacea* is also typical. These grasslands are generally managed by mowing or low‐intensity cattle grazing. Characteristic forb species are *Aster tripolium* subsp. *pannonicus, Cerastium dubium*,* Cirsium brachycephalum, Podospermum canum* and *Rorippa sylvestris* subsp. *kerneri*. Some marsh species like *Cirsium canum, Lysimachia nummularia*,* Lythrum virgatum, Symphytum officinale,* and short‐grass steppe species, such as *Achillea collina, Artemisia pontica* or *Limonium gmelinii* subsp. *hungaricum* are also present (Deák, Valkó, Török, & Tóthmérész, 2014; Figure 2d). Total biomass typically ranges between 800 and 1,000 g/m^2^ (Deák et al., 2015; Kelemen et al., 2013). The soils are wet and compact meadow soils (gleysoils, fluvisoils or vertisoils) with highly varying contents of nutrients but characterized by at least moderate salt contents.

### 2.5 Data collection and analyses

Basic vegetation characteristics (species richness, Shannon diversity, and evenness), and four leaf traits were considered in the analyses. Leaf traits were considered among the most sensitive indicators of grazing in relation to different intensity regimes (Kechang, Meisser, He, & Lechowicz, 2015; Zheng, Li, Lan, Ren, & Wang, 2015); thus, we selected the most frequently measured and analyzed life traits for the analysis. The studied leaf traits were leaf dry matter content (LDMC), leaf dry weight (LDW), leaf area (LA) and specific leaf area (SLA). The leaf trait scores were obtained either (a) from the LEDA trait database (Kleyer et al., 2008), from the (b) publication of Lhotsky et al. (2016) or (c) we used own measurements of species originating from the region, using standardized measurement protocols (Cornelissen et al., 2003). We calculated community‐weighted means (CWMs) for each trait, (Pla, Casanoves, & Di Rienzo, 2012; Villéger et al., 2008). As suggested first by Mason, Mouillot, Lee, and Wilson (2005), we calculated the three components of functional diversity, multi‐trait functional richness (FRic), functional evenness (FEve) and divergence (FDiv) for all studied leaf traits. For the calculation of all of the indices, we used the FDiversity program package. We used Euclidean distances based on species cover scores for weighting (Casanoves, Pla, & Di Rienzo, 2011). Nomenclature for species follows Király (2009).

The effects of “grazing intensity,” “grassland type” (fixed factors) and their interaction on the vegetation characteristics were tested by Generalized Linear Mixed Models (GLMM; Zuur, Ieno, Walker, Saveliev, & Smith, 2009) in SPSS 20.0. “Site” (representing the nestedness of plots) and “year” (humidity as ordinal variable, that is, dry, average, and humid) were included as random factors. Dependent variables were the following: CWMs of FRic, FEve, FDiv, SLA, LDMC, LA, LDW, species richness, Shannon diversity, and evenness. We used Fisher's Least Significant Difference (LSD) to find the significant differences. Models were fitted assuming normally distributed errors using the identity link function of SPSS 20.0.

## 3 RESULTS

**Figure 3:**
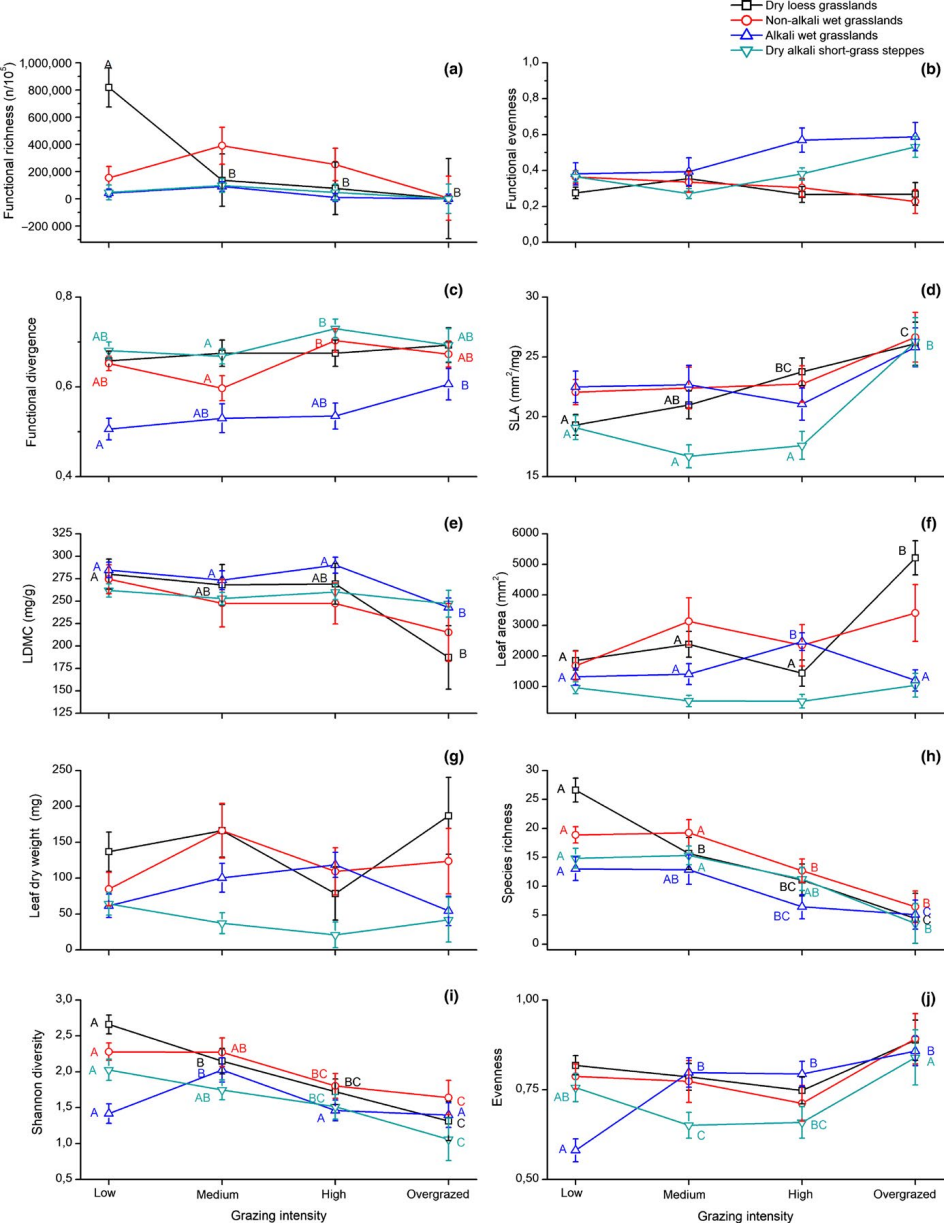
Effect of grazing intensity on the species diversity and functional characteristics of the studied grasslands (CWM ± *SE*). Significant differences between grazing intensity classes (one‐way GLMM and LSD test, *p *< 0.05) were marked with different letters

We found that functional richness was significantly affected both by grassland type and grazing intensity (Table 1). The highest functional richness was detected in low‐intensity grazed dry loess steppes (Figure 3a). The interaction between grassland type and grazing intensity was also significant: functional richness displayed a marked decrease with increasing grazing intensity in dry loess steppes, and in the other three grassland types displayed a humped‐shaped relationship with the highest scores at the medium intensity grazing (Table 1, Figure 3a). Functional evenness was not affected (Figure 3b), while FDiv was significantly affected by grazing intensity (Table 1). Both FEve and FDiv were significantly affected by the grassland type. In the case of FEve, the differences between grassland types became marked at high and overgrazed grasslands, whereas the opposite was found for FDiv where the differences between grassland types almost disappeared at the overgrazed situation (Supporting information Table S2).

**Table 1:** Effect of grazing intensity and grassland type on species diversity and functional characteristics. Generalized mixed effects model with “grazing intensity” and “grassland types” included as fixed factors and “site code” as random factor

Characteristic	Grazing intensity		Grassland type		Interaction	
	*F*_3,606_	*p*	*F*_3,606_	*p*	*F*_9,606_	*p*
Multi‐trait index						
Functional richness	5.816	0.001	5.512	0.001	5.637	<0.001
Functional evenness	1.280	n.s.	10.567	<0.001	3.234	0.001
Functional divergence	3.610	0.013	24.183	<0.001	0.948	n.s.
Community‐weighted mean (CWM)						
SLA	11.372	<0.001	7.319	0.001	1.861	n.s.
LDMC	4.675	0.003	0.690	n.s.	0.658	n.s.
Leaf area	4.522	0.004	18.377	<0.001	3.555	<0.001
Leaf dry weight	1.032	n.s.	9.224	<0.001	1.356	n.s.
Species richness	18.410	<0.001	3.045	0.028	2.102	0.028
Shannon diversity	10.856	<0.001	5.117	0.002	1.662	n.s.
Evenness	6.166	<0.001	2.941	0.033	2.047	0.032

An upward trend of SLA was detected in all grasslands with the highest scores for the overgrazed sites. The change in SLA was also grassland type dependent; in low‐intensity grazed sites a clear separation of grasslands was detected, where the highest SLA was detected in non‐alkali wet grasslands and the lowest in dry alkali short‐grass steppes (Figure 3d and Supporting Information Table S2). LDMC was not affected by the grassland type; a decreasing tendency with increasing grazing intensity was typical in all grasslands, with significantly lower scores in the overgrazed than in the less intensively grazed dry loess steppes and alkali wet grasslands (Figure 3e). The grassland type dependent differentiation decreased with increasing intensity and there was almost no effect detected in the overgrazed grasslands in LDMC (See Supporting Information Table S2). The values for LA and LDWs displayed a similar pattern (Figure 3f,g).

Species richness and Shannon diversity were strongly affected by the grazing intensity. A sharp decline of both species richness and Shannon diversity was typical following medium grazing intensity (Table 1, Figure 3h,i). Some additional grassland type‐dependent effects were observed. In dry loess steppes the species richness and Shannon diversity decreased with increasing grazing intensity, while in the other three grassland types, species richness and Shannon diversity were stable or even increased from low to medium grazing intensity (Figure 3i and Supporting Information Table S2). Evenness was not affected by the grassland type, and in both types of alkali grasslands, the highest scores were detected in overgrazed grasslands (Figure 3j).

## 4 DISCUSSION

### 4.1 Habitat‐dependent effects of intensity hypothesis

We confirmed our hypothesis that the effect of grazing intensity on species and functional diversity was strongly habitat dependent. All species and functional diversity metrics were significantly affected by the grassland type, except LDMC and evenness. The effect of interaction between grazing intensity and grassland type was also proved significant for some metrics (Table 1). This means that the magnitude of the effect differed for most characteristics. In some cases, the trends caused by increasing grazing intensity differed between the subjected grassland types.

The above‐mentioned results were, however, strongly influenced by the different species pools of the grassland types. It is clearly based on the concept of habitat‐specific species pools (Helm, Zobel, Moles, Szava‐Kovats, & Pärtel, 2015), that different grasslands have different sets of characteristic species. In our case, loess grasslands had at least two to three times higher number of characteristic species, which can potentially establish in suitable habitat conditions than the alkali wetlands. This was also expressed in the species richness for the low‐intensity grazed plots. Thus, the differences between grassland types were strongly linked to the different species pools of grasslands subjected to grazing.

Liu et al. (2015) suggested that the effects of grazing are more likely driven by the diversity of the vegetation than by biomass at the small scale. By analyzing sheep grazing in various types of grasslands, Mládek et al. (2011) found that the diet selection of the grazer was strongly influenced by the assembly of traits in the subjected vegetation. Thus, when high forage value species are abundant and the grazing intensity is low, the grazers tend to maximize forage quality of their intake and express a high diet selection (Liu et al., 2015). While the highest number of species, the highest diversity and functional diversity was found for loess grasslands under low‐intensity grazing, these scores rapidly decreased to the level of other grassland types with the increase in grazing intensity from low to medium, supporting previous findings. Loess steppe grasslands are among the most remarkable and valuable grasslands in the steppe zone harboring high species diversity especially for forbs (Török, Kelemen, et al., 2011; Wesche et al., 2016). Our results demonstrated that this type of grasslands was very sensitive to even a slight change in the disturbance regime, that is, a relatively slight increase in grazing intensity can cause dramatic changes in their species pool and functional diversity. Kelemen et al. (2013) found that species richness in an alkaline landscape harboring several types of grasslands along a broad biomass gradient displayed a unimodal relationship with biomass, and the highest species richness was found in loess grasslands with an intermediate level of biomass. It has been suggested that these communities will respond to any change in management, such as from traditional low‐intensity mowing or grazing to abandonment or intensification, by a decrease in species richness (Kelemen et al., 2013). Our results support this theory and are also well in accordance with the intermediate disturbance hypothesis (Connell, 1978) reviewed by Dengler et al. (2014) as another likely explanation of the humped‐back relationship between biomass and species richness.

### 4.2 Intensity‐dependent selectivity hypothesis

Grazing is considered as a selective disturbance which decreases the magnitude of interspecific competition by shifting the trait pool toward to herbivory resistance/avoidance (Carmona et al., 2012; Peco et al., 2017). Diet selectivity in grazing refers mostly to the selection of fodder with higher nutritive value and less fiber tissues, that is, for species with high SLA and low LDMC, or thin and soft leaves (Mládek et al., 2013). There is, however, a trade‐off between the intake of preferred fodder and time/energy it takes to find it in appropriate amounts (Mládek et al., 2013). Thus, by increasing grazing intensity via increased stocking rates, the availability of high quality fodder decreases, resulting in an increased likeliness of selecting lower quality fodder and a decreased rate of diet selectivity.

In line with the comparison by Tóth et al. (2018) of sheep and cattle grazing in short‐grass steppes, our results validated that the increase in grazing intensity decreases the selectivity of grazers, expressed in the increase of SLA. It must be noted that the mentioned effects were only marked in cases of overgrazing compared to the other grazing intensity levels. This can be explained by the general grazing habit of cattle, and thus, its magnitude is highly grazer dependent. Jerrentrup et al. (2015) and Rook et al. (2004) reported that cattle was less selective to forbs compared to sheep, and cattle, in general, was more likely characterized by a “maximising intake” strategy (see Mládek et al., 2013); therefore, selecting patches with higher biomass instead of selecting for individual species, that is, with lower SLA (see also Török, Valkó, Deák, Kelemen, & Tóthmérész, 2014). This behavior suggests that in cases of low and medium density cattle grazing, cattle likely suppress the dominant species of the respective habitat, in most cases characteristic graminoid species, causing an increase in functional diversity (Török et al., 2016). Our study confirmed this effect in three different grassland communities: wet alkali and wet nonalkali grasslands, and dry alkali short‐grass steppes characterized by the dominance of a single or several graminoid species. The functional diversity displayed a unimodal relationship with a peak at medium intensity grazing; while the Shannon diversity and species richness remained stable at low and medium grazing intensity or displayed also a unimodal curve with a drop of the figures at high grazing intensity. It also has to be noted that increasing grazing pressure favors species with fast resource acquisition, that is, those species which produce biomass and grow leaves rapidly and are characterized by high SLA. In contrast, when grazing intensity is low then species with an effective resource conservation and a long leaf lifespan are favoured (i.e., species with high LDMC) (Garnier, Shipley, Roumet, & Laurent, 2001; Kazakou et al., 2007; Poorter & Garnier, 1999).

### 4.3 Intensity‐dependent evenness hypothesis

We hypothesized that higher grazing intensity increases evenness and FEve of the communities. This hypothesis was only partly supported by our findings. We found that evenness was affected but FEve was not affected by grazing intensity. FEve scores, however, were more related to the grassland type (displayed a high divergence) than to the changes in grazing intensity. The two types of alkali grasslands displaying the lowest functional richness showed an increased evenness, while evenness decreased in the other two communities. In both alkali grassland types, the low functional richness (associated also with low species richness) is caused mostly by the high dominance of some graminoids which were likely suppressed by grazing. The suppression of the competitor species by grazing can lead to three well‐documented benefits: (a) direct decrease in species competition by the suppression of the dominant competitor (Török et al., 2014) (b) influencing the light availability near to the soil surface and opening vegetation gaps for colonization (Rook et al., 2004) and (c) increasing the establishment success of zoochorous species transferred by livestock (Ozinga et al., 2009). Surprisingly, we did not detect an increase in species richness, species diversity or functional diversity in the subjected alkali grasslands. This is most likely due to the limited species pool of alkali grasslands, where only a limited number of species can establish (Török et al., 2016). The evenness pattern of alkali grasslands was influenced but neither their richness nor their abundance was likely increased by greater grazing intensity.

## 5 CONCLUSIONS

Our results indicated that the effects of grazing intensity were strongly grassland dependent. We stressed that there is no single management strategy which can be applied to all grasslands; rather, actions should be fine‐tuned at least at the level of grassland type. In this study, we found that out of the four typical grassland types of steppe zone, the species‐rich loess grasslands on chernozem soils were the most vulnerable and their species richness and functional diversity decreased the most rapidly even with the slightest increase in management intensity. Thus, we stress that the management and conservation of these types of grasslands need the most careful management planning. Based on our results, only low‐intensity grazing should be recommended when grazing management is planned. In the other three grassland types, species richness and diversity remained stable or in some of the grasslands even increased from low to medium grazing intensity. Thus, the vegetation of the other three studied grassland types may tolerate medium grazing intensity of cattle without significant decrease in species richness and diversity.

## CONFLICT OF INTEREST

The authors declare no conflict of interest.

## AUTHOR CONTRIBUTIONS

PT and BT conceived the ideas and designed methodology; KP and ET collected the data; PT, AK, and BT analyzed the data; PT led the writing of the manuscript with substantial contribution of JS. All authors contributed critically to the drafts and gave final approval for publication.

## DATA ACCESSIBILITY

In case of acceptance, the authors upload all primary data of the manuscript to the Dryad Digital Repository.
