# Supplementary material for: Vegetation type and grazing intensity jointly shape grazing effects on grassland biodiversity
Source: Ecol Evol. 2018 Oct 3;8(20):10326–35. doi: 10.1002/ece3.4508 (PMC6206222; doi:10.1002/ece3.4508)
Supplement: Supplementary file 1 [file ECE3-8-10326-s001.doc]

**Supporting Information Table S1.** Study sites. Notations: Grazing intensity = L-Low (<1.0 Animal unit per ha – AU/ha); M-Medium (1.0-2.5 AU/ha); H-high (3.0-8.0 AU/ha); O-Overgrazed (≥ 20.0 AU/ha). Grassland types: AG - Dry alkali short-grass steppes; LG – Dry loess steppes; WG – Non-alkali wet grasslands; AWG – Alkali wet grasslands.

| **Site code** | **Grassland type** | **Number of plots** | **Grazing intensity** | **Nearest settlement** | **Year of sampling** |
| --- | --- | --- | --- | --- | --- |
| S01 | LG | 5 | L | Dejtár | 2016 |
| S02 | LG | 7 | L | Csákvár | 2015 |
| S03 | LG | 6 | L | Szabadszállás | 2015 |
| S04 | LG | 5 | L | Tihany | 2002 |
| S05 | LG | 10 | L | Tihany | 2002 |
| S06 | LG | 5 | L | Tihany | 2007 |
| S07 | LG | 5 | L | Tihany | 2008 |
| S08 | LG | 5 | L | Tihany | 2010 |
| S09 | LG | 6 | L | Keszthely | 2007 |
| S10 | LG | 10 | M | Gyulakeszi | 2008 |
| S11 | LG | 5 | M | Csopak | 2007 |
| S12 | LG | 5 | M | Dejtár | 2016 |
| S13 | LG | 6 | M | Szabadszállás | 2015 |
| S14 | LG | 5 | M | Csopak | 2007 |
| S15 | LG | 5 | H | Bugac | 2005 |
| S16 | LG | 5 | H | Kunbaracs | 2007 |
| S17 | LG | 5 | H | Tatárszentgyörgy | 2007 |
| S18 | LG | 5 | H | Kunbaracs | 2007 |
| S19 | LG | 10 | H | Káptalantóti | 2007 |
| S20 | LG | 10 | O | Csopak | 2005 |
| S21 | LG | 10 | O | Tihany | 2012 |
| S22 | WG | 5 | L | Tatárszentgyörgy | 2007 |
| S23 | WG | 5 | L | Tatárszentgyörgy | 2007 |
| S24 | WG | 8 | L | Viszló | 2009 |
| S25 | WG | 15 | L | Beregdaróc | 2012 |
| S26 | WG | 5 | L | Kunbaracs | 2007 |
| S27 | WG | 5 | L | Kunbaracs | 2007 |
| S28 | WG | 15 | L | Gyulakeszi | 2007 |
| S29 | WG | 5 | L | Csokvaomány | 2007 |
| S30 | WG | 5 | M | Balatoncsicsó | 2010 |
| S31 | WG | 5 | M | Gyulakeszi | 2007 |
| S32 | WG | 10 | M | Csákvár | 2016 |
| S33 | WG | 5 | H | Gyulakeszi | 2007 |
| S34 | WG | 5 | H | Badacsonytördemic | 2007 |
| S35 | WG | 4 | H | Keszthely | 2007 |
| S36 | WG | 10 | H | Csákvár | 2016 |
| S37 | WG | 10 | O | Gyulakeszi | 2005 |

**Supporting Information Table S1.** Continued

| **Site code** | **Grassland type** | **Number of plots** | **Grazing intensity** | **Nearest settlement** | **Year of sampling** |
| --- | --- | --- | --- | --- | --- |
| S38 | WG | 10 | O | Badacsonytördemic | 2005 |
| S39 | AWG | 13 | L | Biharugra | 2012 |
| S40 | AWG | 15 | L | Hortobágy | 2015 |
| S41 | AWG | 15 | L | Hortobágy | 2015 |
| S42 | AWG | 10 | M | Vésztő | 2002 |
| S43 | AWG | 13 | M | Vésztő | 2006 |
| S44 | AWG | 3 | H | Biharugra | 2012 |
| S45 | AWG | 15 | H | Szabadszállás | 2014 |
| S46 | AWG | 10 | H | Kisványon | 2005 |
| S47 | AWG | 10 | O | Biharugra | 2005 |
| S48 | AWG | 10 | O | Tatárszentgyörgy | 2012 |
| S49 | AG | 10 | L | Hortobágy | 2014 |
| S50 | AG | 10 | L | Ballószög | 2007 |
| S51 | AG | 10 | L | Bugac | 2005 |
| S52 | AG | 8 | L | Bugac | 2005 |
| S53 | AG | 6 | L | Solt | 2007 |
| S54 | AG | 10 | L | Hódmezővásárhely | 1997 |
| S55 | AG | 12 | L | Hódmezővásárhely | 2005 |
| S56 | AG | 10 | L | Egyek | 2014 |
| S57 | AG | 10 | M | Egyek | 2014 |
| S58 | AG | 10 | M | Hortobágy | 2014 |
| S59 | AG | 10 | M | Hortobágy | 2014 |
| S60 | AG | 10 | M | Hortobágy | 2014 |
| S61 | AG | 12 | M | Szabadszállás | 2015 |
| S62 | AG | 10 | M | Hortobágy | 2014 |
| S63 | AG | 10 | M | Hortobágy | 2014 |
| S64 | AG | 10 | M | Hortobágy | 2014 |
| S65 | AG | 10 | M | Hortobágy | 2014 |
| S66 | AG | 10 | H | Kunmadaras | 2014 |
| S67 | AG | 10 | H | Kunmadaras | 2014 |
| S68 | AG | 10 | H | Kunmadaras | 2014 |
| S69 | AG | 10 | H | Kunmadaras | 2014 |
| S70 | AG | 9 | H | Solt | 2007 |
| S71 | AG | 10 | H | Hortobágy | 2014 |
| S72 | AG | 10 | O | Szabadszállás | 2010 |
| S73 | AG | 9 | O | Kunbaracs | 2010 |
