# Supplementary material for: Vegetation type and grazing intensity jointly shape grazing effects on grassland biodiversity
Source: Ecol Evol. 2018 Oct 3;8(20):10326–35. doi: 10.1002/ece3.4508 (PMC6206222; doi:10.1002/ece3.4508)
Supplement: Supplementary file 2 [file ECE3-8-10326-s002.doc]

**Supporting Information Table S2.** Effect of grassland type on the species diversity and functional characteristics of the studied grasslands. Significant differences (One-way GLMM and LSD test, p<0.05) were marked with different letters. For the means see Figure 1. Notations: Grazing intensity = L-Low (<1.0 Animal unit per ha – AU/ha); M-Medium (1.0-2.5 AU/ha); H-high (3.0-8.0 AU/ha); O-Overgrazed (≥ 20.0 AU/ha). Grassland types: AG - Dry alkali short-grass steppes; LG – Dry loess steppes; WG – Non-alkali wet grasslands; AWG – Alkali wet grasslands.

| **Functional richness** | | | | | |  | **Functional divergence** | | | | | | |
| --- | --- | --- | --- | --- | --- | --- | --- | --- | --- | --- | --- | --- | --- |
|  |  | Grassland type | | | |  |  |  | Grassland type | | | | |
|  |  | **LG** | **WG** | **AWG** | **AG** |  |  |  | **LG** | **WG** | **AWG** | **AG** |  |
| Grazing intensity | L | A | B | B | B |  | Grazing intensity | **L** | A | A | B | A |  |
|  | M | x | x | x | x |  |  | **M** | A | AB | B | A |  |
|  | H | x | x | x | x |  |  | **H** | A | A | B | A |  |
|  | O | x | x | x | x |  |  | **O** | x | x | x | x |  |
|  |  |  |  |  |  |  |  |  |  |  |  |  |  |
| **Functional evenness** | | | | | |  | **Specific Leaf Area** | | | | | | |
|  |  | Grassland type | | | |  |  |  | Grassland type | | | | |
|  |  | **LG** | **WG** | **AWG** | **AG** |  |  |  | **LG** | **WG** | **AWG** | **AG** |  |
| Grazing intensity | **L** | x | x | x | x |  | Grazing intensity | **L** | AB | C | BC | A |  |
|  | **M** | x | x | x | x |  |  | **M** | A | A | A | B |  |
|  | **H** | A | A | B | A |  |  | **H** | A | A | AB | B |  |
|  | **O** | A | A | B | B |  |  | **O** | x | x | x | x |  |
|  |  |  |  |  |  |  |  |  |  |  |  |  |  |
| **Leaf Dry Matter Content** | | | | | |  | **Leaf area** | | | | | | |
|  |  | Grassland type | | | |  |  |  | Grassland type | | | | |
|  |  | **LG** | **WG** | **AWG** | **AG** |  |  |  | **LG** | **WG** | **AWG** | **AG** |  |
| Grazing intensity | **L** | x | x | x | x |  | Grazing intensity | **L** | x | x | x | x |  |
|  | **M** | x | x | x | x |  |  | **M** | A | A | AB | B |  |
|  | **H** | x | x | x | x |  |  | **H** | A | B | B | C |  |
|  | **O** | x | x | x | x |  |  | **O** | A | AB | B | B |  |
|  |  |  |  |  |  |  |  |  |  |  |  |  |  |
|  |  |  |  |  |  |  |  |  |  |  |  |  |  |
| **Leaf dry weight** | | | | | |  | **Species richness** | | | | | | |
|  |  | Grassland type | | | |  |  |  | Grassland type | | | | |
|  |  | **LG** | **WG** | **AWG** | **AG** |  |  |  | **LG** | **WG** | **AWG** | **AG** |  |
| Grazing intensity | **L** | A | AB | B | B |  | Grazing intensity | **L** | A | B | B | B |  |
|  | **M** | A | AB | AB | B |  |  | **M** | x | x | x | x |  |
|  | **H** | A | A | A | B |  |  | **H** | x | x | x | x |  |
|  | **O** | A | AB | B | B |  |  | **O** | A | B | A | C |  |
|  |  |  |  |  |  |  |  |  |  |  |  |  |  |
| **Shannon diversity** | | | | | |  | **Evenness** | | | | | | |
|  |  | Grassland type | | | |  |  |  | Grassland type | | | | |
|  |  | **LG** | **WG** | **AWG** | **AG** |  |  |  | **LG** | **WG** | **AWG** | **AG** |  |
| Grazing intensity | **L** | A | AB | C | BC |  | Grazing intensity | **L** | A | A | B | A |  |
|  | **M** | x | x | x | x |  |  | **M** | A | AB | AB | B |  |
|  | **H** | x | x | x | x |  |  | **H** | x | x | x | x |  |
|  | **O** | A | B | A | C |  |  | **O** | A | A | AB | B |  |
